# Cyclophilin A Interacts with Viral VP4 and Inhibits the Replication of Infectious Bursal Disease Virus

**DOI:** 10.1155/2015/719454

**Published:** 2015-05-24

**Authors:** Nian Wang, Lizhou Zhang, Yuming Chen, Zhen Lu, Li Gao, Yongqiang Wang, Yulong Gao, Honglei Gao, Hongyu Cui, Kai Li, Changjun Liu, Yanping Zhang, Xiaole Qi, Xiaomei Wang

**Affiliations:** ^1^Division of Avian Infectious Diseases, State Key Laboratory of Veterinary Biotechnology, Harbin Veterinary Research Institute, The Chinese Academy of Agricultural Sciences, Harbin 150001, China; ^2^Jiangsu Co-Innovation Center for Prevention and Control of Important Animal Infectious Disease and Zoonoses, Yangzhou 225009, China

## Abstract

Nonstructural protein VP4, a serine protease of infectious bursal disease virus (IBDV) that catalyzes the hydrolysis of polyprotein pVP2-VP4-VP3 to form the viral proteins VP2, VP4, and VP3, is essential to the replication of IBDV. However, the interacting partners of VP4 in host cells and the effects of the interaction on the IBDV lifecycle remain incompletely elucidated. In this study, using the yeast two-hybrid system, the putative VP4-interacting partner cyclophilin A (CypA) was obtained from a chicken embryo fibroblast (CEF) expression library. CypA was further confirmed to interact with VP4 of IBDV using co-immunoprecipitation (CO-IP), GST pull-down, and confocal microscopy assays. Moreover, we found that the overexpression of CypA suppressed IBDV replication, whereas the knock-down of CypA by small interfering RNAs promoted the replication of IBDV. Taken together, our findings indicate that the host cell protein CypA interacts with viral VP4 and inhibits the replication of IBDV.

## 1. Introduction

Infectious bursal disease (IBD), which was first described as Gumboro disease, is an acute, highly contagious disease in young chickens that causes significant economic losses in the poultry industry worldwide [1]. Infectious bursal disease virus (IBDV), the causative agent of IBD, targets the bursa of Fabricius, leading to severe immunosuppression by destroying B lymphocytes, attracting T cells and activating macrophages [2, 3].

IBDV is a member of the genus* Avibirnavirus* belonging to the* Birnaviridae* family [4]. Its genome consists of two segments of double-stranded RNAs (A and B) [5]. Segment A contains two partially overlapping open reading frames (ORFs) [6]. The first ORF encodes a 17 kD nonstructural viral protein denoted VP5, which has been implicated in the induced bursal pathology and the egress of the virus from infected cells [7, 8]. The second ORF encodes a 110 kD polyprotein (pVP2-VP4-VP3 precursor) that can be autocatalytically cleaved into two structural proteins (VP2 and VP3) and a serine protease (VP4) [9, 10]. VP2 is the major structural and virulence protein and can elicit the neutralizing antibodies [11, 12]. VP3, a group special and immunogenic protein of IBDV, interacts with VP1, which is encoded by segment B, and binds to the viral dsRNA to form ribonucleoprotein complexes [13]. VP1, a RNA-dependent RNA polymerase (RdRp), acts as a genome-linked protein and cyclizes segments A and B [14].

Nonstructural protein VP4 catalyzes the hydrolysis of polyprotein pVP2-VP4-VP3 to form the viral proteins VP2, VP4, and VP3 utilizing the serine-lysine (Ser-652 and Lys-692) catalytic dyad in the active site, which has* trans* activity [15, 16]. The cleavage sites of pVP2-VP4 (511LAA513) and VP4-VP3 (754MAA756) have been established [17]. VP4 obviously plays a key role in the maturation of IBDV. Moreover, it has been reported that the glucocorticoid-induced leucine zipper protein (GILZ) of the host cell is hijacked by VP4 to enhance IBDV growth [18]. In this study, we first identified that the host protein cyclophilin A (CypA) is a novel interacting partner of IBDV VP4 and may inhibit the replication of IBDV.

## 2. Materials and Methods

### 2.1. Cells and Viruses

DF-1 cells (immortal chicken embryo fibroblasts) and HEK293T cells, obtained from the American Type Culture Collection (ATCC), were grown in Dulbecco's modified Eagle medium (DMEM; Invitrogen, USA) containing 10% fetal bovine serum (FBS). Chicken embryo fibroblast (CEF) cells were prepared from 10-day-old specific-pathogen-free (SPF) chicken embryos and cultured in DMEM supplemented with 4% FBS. Both of these cells were grown at 37°C in a 5% CO_2_ incubator. The IBDV Gt strain was prepared in our laboratory as described previously [19].

### 2.2. Plasmids

The VP4 gene of IBDV was amplified from the Gt strain (GenBank accession number: DQ403248) and cloned into the yeast expression vector pGBKT7, denoted as the bait plasmid pGB-VP4, and the eukaryotic expression vector pCAGGS-HA, which includes a HA-tag at the N terminus and is named pCAH-VP4. The CypA gene of chicken (GenBank accession number: NM_001166326), which was amplified from CEF by RT-PCR, was fused to the prokaryotic expression vector pGEX-6p-1, denoted as pGEX-CypA, and the eukaryotic expression vector pCAGGS-Flag, which includes a Flag-tag at the C terminus and was named pCAF-CypA. All of the primers and restriction enzyme sites are listed in Table 1.

### 2.3. Yeast Two-Hybrid Screen

The potential host proteins interacting with VP4 were screened using the Matchmaker Gold Yeast Two-Hybrid System (Cat. number 630489, Clontech). The cDNA library of CEF cells was cloned in pGADT7 and transformed into the yeast strain Y187 using the Mate and Plate Library System (Cat. number 630490, Clontech). The bait plasmid pGB-VP4 was transformed into the yeast strain Y2H, named Y2H/BD-VP4. The self-activation and virulence were tested using the protocol described in the Matchmaker Gold Yeast Two-Hybrid System User Manual (PT4084-1, Clontech). Y2H/BD-VP4 was hybridized with the cDNA library, and the hybrid yeasts were then cultured on SD plates lacking Leu and Trp (SD/-Leu/-Trp, DDO). The colonies were then transferred to quadruple-dropout plates lacking His, Leu, Trp, and Ade (SD/-4, QDO), and the positive clones with a blue color were then selected on SD/-4 plates containing X-*α*-Gal and aureobasidin A (QDO/X/A). The plasmids of the positive clones were isolated according to the Product Manual of the Yeast Plasmid Kit (D3376-02, OMEGA), amplified in* E. coli* DH5*α* and sequenced (primers for pGADT7 are shown in Table 1). To confirm the positive results, we cotransformed the prey plasmids and the bait plasmid pGB-VP4 into the yeast strain Y2H and retested them on DDO, QDO, and QDO/X/A. In addition, a positive control (cotransformed with pGBKT7-53 and pGADT7-T), negative control (cotransformed with pGBKT7-Lam and pGADT7-T), and blank control (cotransformed with pGBKT7 and pGADT7) were also included.

### 2.4. Co-Immunoprecipitation (CO-IP) and Western Blot

HEK293T cells were cultured in a monolayer to 80–90% in six-well plates. The pCAH-VP4 and pCAF-CypA were then transfected into HEK293T cells together or alone using Lipofectamine 2000 (Invitrogen, USA) according to the manufacturer's instructions. Empty vectors were used as controls. At 48 h after transfection, the supernatants were discarded, and the cells were washed three times with cold PBS. Two hundred microliters of cold Western and Immunoprecipitation Lysis Buffer (Beyotime Institute of Biotechnology, Beijing, China) containing 1 mM phenylmethylsulfonyl fluoride (PMSF) (Beyotime Institute of Biotechnology, Beijing, China) was added to the cells, and the cells were then incubated for 30 min at 4°C. The mixture lysates were transferred to cold tubes and centrifuged at 15,000 ×g and 4°C for 10 min. The supernatants were then transferred to new tubes containing protein A/G beads (Beyotime Institute of Biotechnology, Beijing, China) and incubated for 2 h at 4°C. After centrifugation of the precleared lysates, new protein A/G beads were added to the lysates for another 4 h at 4°C. Finally, 160 *μ*L of the supernatant lysates was incubated with the anti-HA monoclonal antibody (mAb) (H9658, sigma) for 4 h at 4°C. The rest of the 40 *μ*L supernatants were used as the input control. The bead complexes were washed three times with 1 mL of cold PBS, boiled in 5x SDS-PAGE sample loading buffer, subjected to 12% SDS-PAGE gel, immunoblotted with anti-Flag (F1804, Sigma) and anti-HA mAb, and then incubated with IRDye 800CW goat anti-mouse IgG (LiCor BioSciences, Lincoln, NE, USA). The blots were detected using the Odyssey Infrared Imaging System (LiCor BioSciences, Lincoln, NE, USA).

### 2.5. GST Pull-Down Assay

The pGEX-CypA plasmid was transformed into* E. coli* BL21 for the GST pull-down assay. GST-CypA and GST proteins were conjugated to the glutathione beads (GE Healthcare) and blocked for 1 h in 5% BSA. After washing three times with TIF buffer (20 mM Tris-HCl [pH 8.0], 150 mM NaCl, 1 mM MgCl_2_, 0.1% NP-40, 10% glycerol, 0.1 mM dithiothreitol [DTT], and 1 mg/mL protease inhibitor), the beads were incubated with the cell lysates containing the HA-VP4 proteins expressed by HEK293T cells transfected with pCAH-VP4 for 4 h at 4°C. The beads were then washed six times with TIF buffer and detected by western blot. At the same time, 40 *μ*L of the cell lysates expressing HA-VP4 was used to show the presence of the VP4 protein by western blot. First, anti-HA mAb was used to detect the HA-VP4 protein, and the anti-GST polyclonal antibody (G7781, Sigma) was then used to detect the GST or GST-CypA. IRDye 800CW goat anti-mouse and anti-rabbit IgG (LiCor BioSciences, Lincoln, NE, USA) were used to conjugate the anti-HA mAb and anti-GST polyclonal antibody, respectively. Finally, blots were detected using the Odyssey Infrared Imaging System.

### 2.6. Confocal Laser Scanning Microscopy

DF-1 cells were cotransfected with pCAH-VP4 and pCAF-CypA. Single transfection with pCAH-VP4 or pCAF-CypA was performed as controls. After 48 h, the cells were fixed with 4% paraformaldehyde for 30 min at 37°C, permeabilized with 0.1% Triton X-100 for 10 min, and blocked with 3% bovine serum albumin (BSA). Anti-HA rabbit polyclonal antibody (H6908, Sigma) and anti-Flag mouse mAb (F1804, Sigma) were incubated with the cells for 1 h. After washing five times with PBS, the cells were incubated with the FITC- or TRITC-conjugated secondary antibody (Sigma) for 1 h at 37°C. The cells were then stained with DAPI for 10 min at 37°C and examined using a Leica SP2 Confocal system (Leica Microsystems, Wetzlar, Germany).

### 2.7. CypA Overexpression Assay and Virus Infection

The pCAF-CypA plasmid was transfected into DF-1 cells in six-well plates to overexpress the CypA proteins, and the empty vector pCAF was used as a control. At 24 h after transfection, the cells were washed three times with PBS. The cells were then infected with the IBDV Gt strain (MOI = 0.01) in 2 mL of FBS-free DMEM. After incubation for 1 h at 37°C, the cells were washed three times with PBS and cultured in 2 mL of fresh DMEM with 10% FBS. At 48 h after infection, the cell supernatants were collected to detect the genomic copies of IBDV using real-time RT-PCR and the viral infectivity titers as TCID_50_ per 100 *μ*L. The cells were lysed immediately to detect the expression of VP2 (or pVP2), CypA-Flag, and GAPDH by western blot.

Meanwhile, to evaluate the effects of overexpression of CypA on DF-1 cells, the cell counting kit-8 (CCK-8, DOJINDO) was used to detect the cell viability and proliferation. DF-1 cells were cultured in 96-well plates for 48 h after transient transfection with pCAF-CypA plasmid or the empty vector pCAF. Another group with no treatment was also used as control. Then, 10 *μ*L of CCK-8 solution was added into each well of the plates and incubated the plates for another 6 hours at 37°C. The absorbance was measured at 450 nm using a microplate reader (Gene Company Limted, China).

### 2.8. RNA Interference

siRNAs targeting CypA (GenBank accession number: NM_001166326) were designed and synthesized by Shanghai GenePharma Company (Table 1). To evaluate the knock-down efficiency of the siRNAs, 100 pmol of one of the siRNAs and 1 *μ*g of pCAF-CypA were cotransfected in DF-1 cells. After 24 h, the cells were lysed for western blotting with anti-Flag mAb and anti-*β*-tubulin antibody (T8328, Sigma). The siRNA with the best knock-down effect was chosen for transfecting DF-1 cells. At 24 h after transfection, the siRNA-transfected cells were infected with the IBDV Gt strain at an MOI of 0.01 and cultured for 48 h. The supernatants were then collected to detect the genomic copies and viral titers of IBDV. The cells were lysed to detect the expression of viral VP2 (or pVP2) and GAPDH. Meanwhile, the effects of the knock-down of CypA on the viability and proliferation of DF-1 cells were also evaluated using CCK-8 as described above and DF-1 cells that were transfected with the negative control siRNA (NC) were used as control. Another group with no treatment was also used as control.

### 2.9. Viral RNA Isolation and Real-Time RT-PCR

The RNAs from 50 *μ*L of the IBDV-infected cell supernatants were isolated with the Viral RNA Kit (R6874-02, OMEGA) and dissolved in 100 *μ*L of DEPC water. The RNAs were then used to detect the numbers of the genomic copies of IBDV according to the One Step Prime Script RT-PCR Kit User Manual (RR064A, TaKaRa) and quantified with a Light Cycler 480 II (Roche). The primers and probe are shown in Table 1.

### 2.10. Virus Titer Assay

The virus titers of IBDV were detected as TCID_50_ per milliliter by the Reed-Muench method as described previously [20].

### 2.11. Statistical Analysis

The statistical analyses were performed using the GraphPad Prism 6.0 software. Student's *t*-test and one-way ANOVA were used for comparing the genomic RNA copies and viral titers. A *P* value of less than 0.05 was considered significantly different, and a *P* value of less than 0.01 was considered extremely significantly different.

## 3. Results

### 3.1. CypA Was Screened by Yeast Two-Hybrid Screen

**Figure 1:**
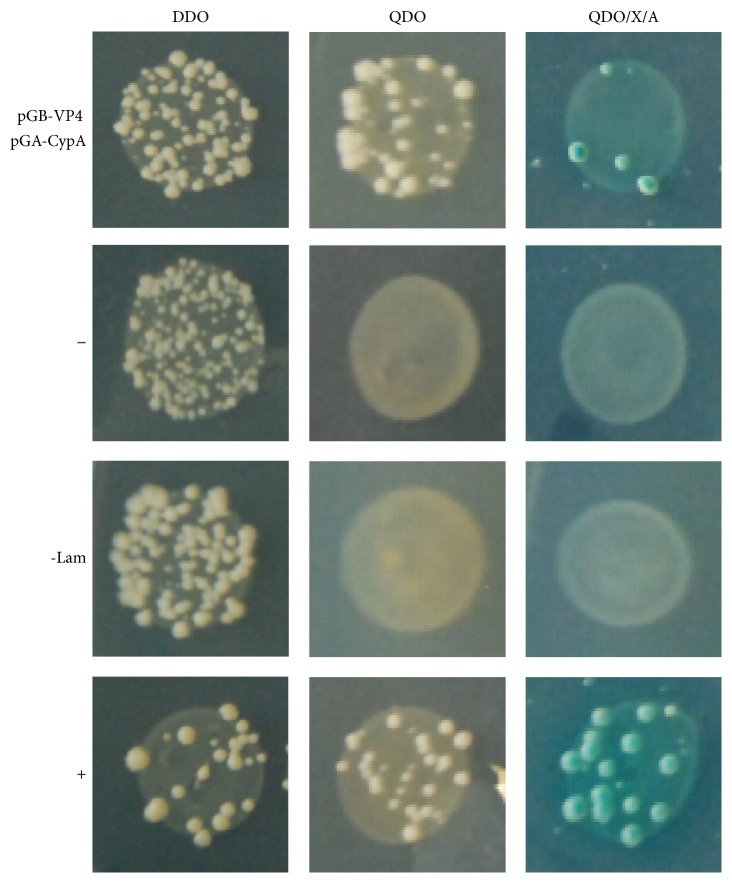
CypA was confirmed to interact with VP4 in yeast. The bait plasmid pGB-VP4 and the prey plasmid pGA-CypA isolated from positive colonies were cotransformed into yeast Y2H strains and retested on DDO, QDO, and QDO/X/A. A positive control (pGBKT7-53 and pGADT7-T, +), negative control (pGBKT7-Lam and pGADT7-T, -Lam), and blank control (pGBKT7 and pGADT7, −) were also included.

To identify the host cellular proteins interacting with VP4 of IBDV, a bait vector pGB-VP4 expressing VP4 of the IBDV strain Gt without self-activation and virulence to yeast cells was constructed. In the initial screen of a cDNA library of CEF cells using the Matchmaker Gold Yeast Two-Hybrid System, 117 putative positive clones were obtained on QDO/X/A, and the plasmids in yeast were then isolated, amplified in* E. coli* DH5*α*, and sequenced. Among them, 18 clones, including CypA, were in frame with the GAL4 DNA-activation domain (data not shown). To confirm the interaction between VP4 and CypA, we transformed the prey plasmid into the yeast Y2H strain in combination with the bait plasmid pGB-VP4 and retested their interaction on DDO, QDO, and QDO/X/A, which yielded positive results (Figure 1).

### 3.2. CypA Interacts with VP4

**Figure 2:**
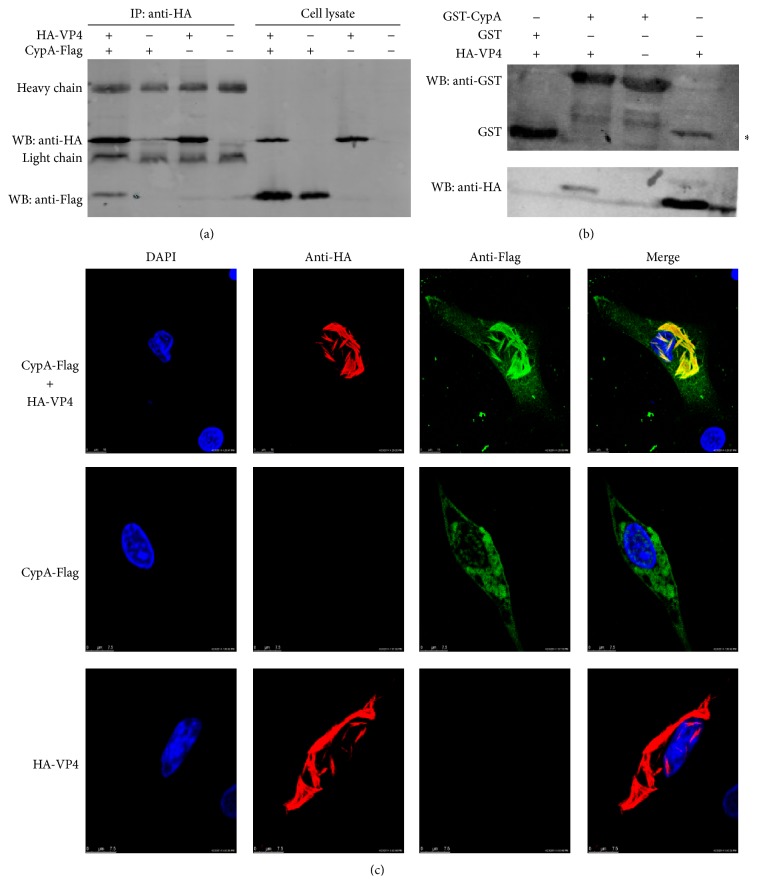
Interaction of IBDV VP4 with CypA. (a) CO-IP of HA-VP4 and CypA-Flag. PCAH-VP4 and pCAF-CypA were transfected into HEK293T cells together or alone. At 48 h after transfection, the cells were lysed for immunoprecipitation (IP) directed by anti-HA mAb. The proteins were detected by western blotting with anti-HA and anti-Flag mAb. Coexpressing HA-VP4 and CypA-Flag can form a complex, whereas neither single HA-VP4 nor CypA-Flag alone could. (b) GST pull-down assay. The soluble protein GST-CypA expressed in* E. coli* BL21 and GST alone were conjugated to glutathione beads and incubated with HEK293T cell lysates expressing the HA-VP4 protein. HA-VP4 was first detected with anti-HA mAb by western blot. GST-CypA or GST was then detected with anti-GST polyclonal antibodies. Cell lysates containing HA-VP4 were used as a control to show VP4 in the last lane. *∗* indicates nonspecific binding. (c) Colocalization of VP4 and CypA. DF-1 cells were cotransfected with pCAH-VP4 and pCAF-CypA. A single transfection was used as a control. At 48 h after transfection, the cells were fixed and subjected to indirect immunofluorescence assays to detect VP4 (red) and CypA (green) with anti-Flag mAb and anti-HA polyclonal antibody. DAPI (blue) was used to indicate the nucleus.

To further identify the interaction between VP4 and CypA, a CO-IP assay was performed. The plasmids expressing HA-VP4 or CypA-Flag were transfected into HEK293T cells together or alone, and empty vectors were used as controls. CO-IP directed by anti-HA mAb showed that coexpressing HA-VP4 and CypA-Flag could form a complex, whereas neither HA-VP4 nor CypA-Flag alone was able to form a complex (Figure 2(a)). In addition, a GST pull-down assay was performed to exclude the possibility that the other cellular proteins indirectly mediated the interaction between VP4 and CypA. As shown in Figure 2(b), the GST-tagged CypA pulled down HA-VP4, but GST could not, indicating that the interaction of VP4 and CypA was due to a direct physical association. To confirm the colocalization and interaction, DF-1 cells were cotransfected with plasmids expressing HA-VP4 and CypA-Flag. Single transfections with the plasmid expressing HA-VP4 or CypA-Flag were performed as controls (Figure 2(c)). The results of confocal laser scanning microscopy assays showed that both HA-VP4 and CypA-Flag were distributed and colocalized in the cytoplasm. Moreover, HA-VP4 could self-assemble into tubule-like particles as described previously [21], and the diffused distribution of CypA-Flag could be attracted by HA-VP4 to form tubule-like particles. Taken together, these findings confirmed that the CypA is an interacting protein of VP4.

### 3.3. Overexpression of CypA Suppresses the Replication of IBDV

**Figure 3:**
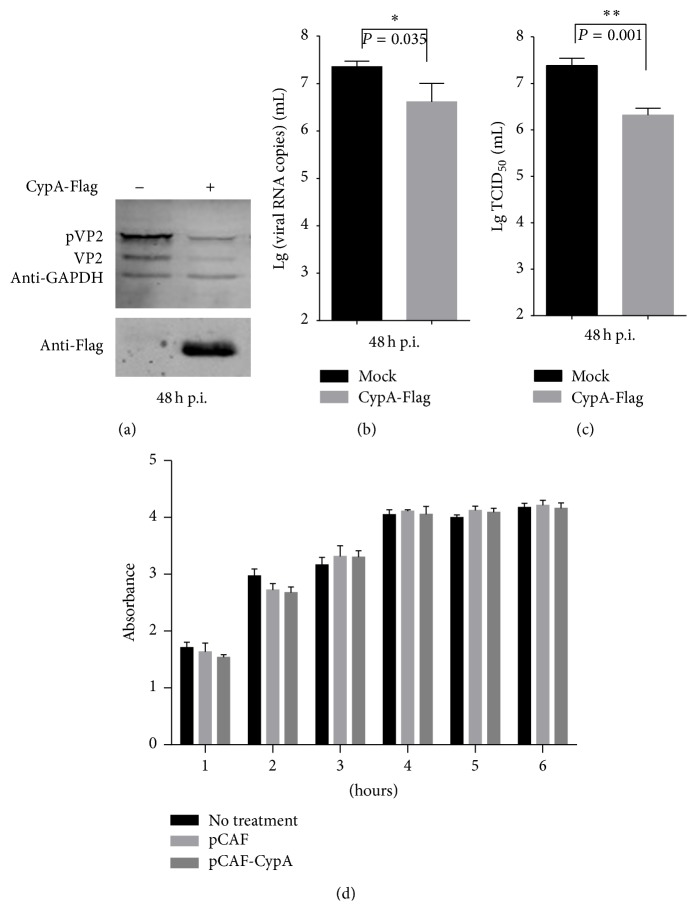
Overexpression of CypA inhibits IBDV replication. (a) The expression of the VP2 and pVP2 proteins was reduced in CypA-overexpressing cells. PCAF-CypA (+) or empty vector (−) was transfected into DF-1 cells for 24 h, and the cells were then infected with the IBDV Gt strain at an MOI of 0.01. At 48 h after infection, the cells were lysed to analyze the effect of the overexpression of CypA on IBDV growth by western blot and to evaluate the expression of IBDV VP2. VP2 (or pVP2) and CypA-Flag were detected with anti-VP2 mAb and anti-Flag mAb, respectively. GAPDH was detected as a loading control. (b) The viral RNA copies were detected through real-time RT-PCR. (c) The virus titers in the culture supernatants of IBDV-infected DF-1 cells were detected as TCID_50_ per milliliter by the Reed-Muench method. (d) The cell viability and proliferation of DF-1 cells were evaluated using CCK-8 kit. The error bars represent the standard errors of the mean from three independent experiments. The *P* values are shown above the bars. ^*∗*^ *P* < 0.05 and ^*∗∗*^ *P* < 0.01 indicate significant differences.

To observe the effect of the upregulation of CypA on IBDV replication, DF-1 cells were transfected with the plasmid expressing CypA-Flag and subsequently infected with IBDV at an MOI of 0.01. At 48 h after infection, the expression of mature VP2 and the pVP2 protein of IBDV was clearly reduced (Figure 3(a)). Compared with the mock group, the numbers of the genomic copies of IBDV RNA in the supernatant of the CypA-overexpressing cells were decreased (Figure 3(b)). Moreover, the viral titers in the supernatant of the CypA-overexpressing cells were still less than those obtained in the mock group (Figure 3(c)). As shown in Figure 3(d), compared with the control of pCAF and no treatment, the absorbance of DF-1 cells transfected with the pCAF-CypA had no significant differences, which indicated that overexpression of CypA did not cause obvious damages to the viability and proliferation of DF-1 cells. All of the examinations suggested that the overexpression of CypA suppresses the replication of IBDV.

### 3.4. Knock-Down of CypA Promotes the Replication of IBDV

**Figure 4:**
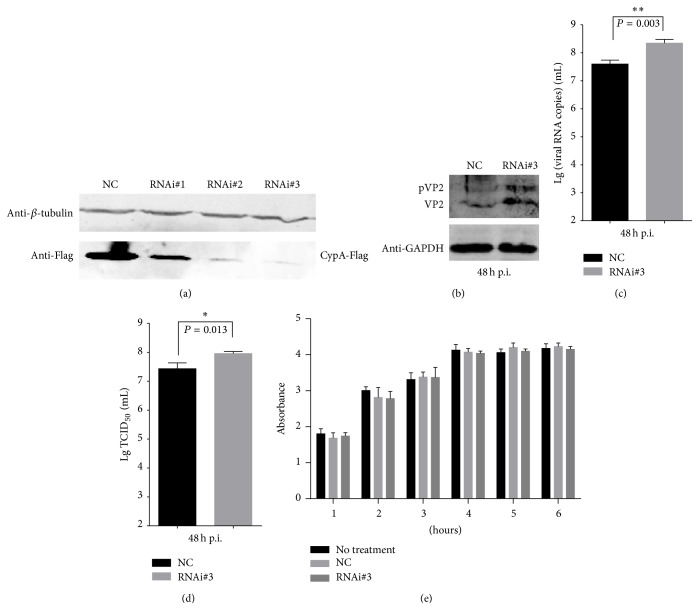
Knock-down of CypA benefits IBDV growth. (a) Interference effect of siRNAs. pCAF-CypA was cotransfected with RNA#1, RNA#2, RNA#3, or negative control (NC) into DF-1 cells. The exogenous CypA expression was used to evaluate the interference effect of the siRNAs. At 24 h after transfection, CypA was detected by western blot with anti-Flag mAb, and *β*-tubulin was used as a loading control. (b) The IBDV VP2 (or pVP2) expression increased in the CypA-knock-down cells. DF-1 cells were treated with RNA#3 or NC for 48 h and then infected with the IBDV Gt strain at an MOI of 0.01 for another 48 h. The cells were harvested to detect IBDV VP2 (or pVP2) by western blot. GAPDH was used as a loading control. (c) The viral RNA copies were detected by real-time RT-PCR. (d) The virus titers in the culture supernatants of IBDV-infected DF-1 cells were detected by the Reed-Muench method. (e) The cell viability and proliferation of DF-1 cells were evaluated using CCK-8 kit. The error bars represent the standard errors of the mean from three independent experiments. The *P* values are shown above the bars. ^*∗*^ *P* < 0.05 and ^*∗∗*^ *P* < 0.01 indicate significant differences.

To further investigate the effect of CypA on the IBDV lifecycle, specific siRNAs targeting CypA in DF-1 cells were synthesized. As shown in Figure 4(a), the transfection of RNA#1, RNA#2, and RNA#3 resulted in the downregulation of exogenous CypA compared with the cells transfected with the negative control siRNA (NC). Because it exhibited the best interference effect, the RNA#3 was used to knock down CypA expression in DF-1 cells. Forty-eight hours after IBDV infection (MOI = 0.01), the expression of mature VP2 and pVP2 proteins of IBDV was significantly increased (Figure 4(b)). Both the genomic copies of IBDV RNA (Figure 4(c)) and the viral titer (Figure 4(d)) in the supernatant of CypA-knock-down cells were higher than obtained for the control cells. As shown in Figure 4(e), the absorbance of DF-1 cells transfected with RNAi#3 had no significant differences with the controls of NC and no treatment, which indicated that knock-down of CypA also did not cause obvious damages to the viability and proliferation of DF-1 cells. Thus, all of the results indicated that the knock-down of CypA promotes the replication of IBDV.

## 4. Discussion

Viruses, unlike other infective agents, cannot proliferate independently without their host cells. They co-opt numerous host proteins to create and maintain a favorable environment for their replication, and the hosts identify the viral infection and stimulate their immune responses to resist viral invasion through the interaction of virus and host proteins. Obviously, the interactions have significant effects on the confrontation between virus and host, helping the host to restrict viral replication and helping the virus utilize the host factors for its own replication. Recent studies have suggested that several host proteins participate in IBDV infection. For example, voltage-dependent anion channel 2 (VDAC2) interacting with VP5 appears to restrict IBDV replication, whereas the receptor of activated protein kinase C 1 (RACK1) binding with VDAC2 and VP5 improves IBDV replication [22, 23]. The other host proteins that are in contact with IBDV proteins, such as heat shock protein 90 (Hsp90), subunit p85*α* of PI3K, and nuclear factor 45 (NF45), also play a critical role in IBDV growth [24–26]. An increased understanding of the relationship of IBDV with the host cells via protein-protein interactions has provided deeper insights into the molecular mechanism underlying IBDV infection.

In this study, the host protein CypA, which interacts with VP4, was screened using the yeast two-hybrid system. Due to the high risk of false positives, the interaction of CypA and VP4 should also be confirmed through additional evidence. CO-IP was performed, and the target protein CypA-Flag was precipitated by the bait protein HA-VP4 with the capture antibody HA mAb. In contrast, HA-VP4 was pulled down with the fusion protein GST-CypA in the GST pull-down assay. Both of these findings further demonstrated the interaction of CypA and VP4. In addition, confocal laser scanning microscopy was used to visualize the intracellular colocalization of CypA and VP4 in cells. VP4 formed tubule-like particles (Figure 2(c)) as reported previously [21, 27]. The distribution of CypA, which is usually diffused in the cytoplasm [28], was changed because of the attraction of VP4. CypA also formed tubule-like particles in the cytoplasm. To explore the effects of CypA on the IBDV lifecycle, the replication of IBDV was then evaluated in cells in which CypA was upregulated or downregulated. The results showed that the overexpression of CypA suppressed IBDV replication, whereas the knock-down of CypA promoted the replication of IBDV. Both of these findings were detected using three different methods, namely, western blot, real-time RT-PCR, and virus titer assay. This study provides the first verification that the host protein CypA interacts with IBDV VP4 and inhibits the replication of IBDV.

CypA is one of the members of the cyclophilin (Cyp) family, which possesses peptidyl-prolyl* cis-trans* isomerase (PPIase) activity [29]. It is an acceleration factor in protein folding and assembly and is also involved in the pathogenesis of cardiovascular disease, cancer, and even viral infection [30–33]. The relationship of CypA and viruses has become a research hotspot. It has been reported that CypA benefits or inhibits viral infections through various mechanisms. For example, CypA is packaged into human immunodeficiency virus (HIV) particles via interacting with the HIV capsid protein Gag and is essential for HIV replication [34]. CypA, interacting with the hepatitis C virus (HCV) protein NS5B, increases the affinity of the polymerase to viral RNA and enhances HCV replication [35]. In addition, CypA incorporates with the matrix protein (M1) of influenza A virus and restricts viral replication by accelerating the degradation of the M1 protein [36, 37]. VP4 is an important protease for IBDV maturation [10]. CypA may be involved in influencing the enzyme function of viral VP4, but the confirmation of this finding requires further studies. In addition, the immune response against IBDV infection does not solely depend on the induction of virus-neutralizing antibody because T cell involvement is also critical. Bursal T cells can be activated and exhibit the upregulation of the gene transcription of proinflammatory cytokines, inducing a so-called “cytokine storm” [38–40]. It has been reported that CypA is involved in T cell activation and in the IFN-I or IL-2 responses during virus infections [41–43]. It is also interesting to explore the function of CypA on the innate immunity against IBDV.

Reports on the interaction of IBDV VP4 and host proteins are few. It was recently reported that the VP4-induced suppression of IFN-I is mediated by the interaction with the host protein GILZ and that this effect benefits the growth of IBDV [18]. In this study, another new interacting partner of IBDV VP4 was identified. Interestingly, the same viral protein (VP4) with different host proteins (GILZ or CypA) had clear different effects on viral replication. The overexpression of CypA blocked the breeding of IBDV, whereas the knock-down of CypA changes the limit of IBDV replication. This finding indicates that IBDV VP4 has multiple functions in the lifecycle of IBDV and that the regulatory mechanism of the virus and host cells through protein-protein interactions is extremely complicated.

## 5. Conclusions

In summary, our study using yeast two-hybrid system, CO-IP, GST pull-down, and confocal laser scanning microscopy assays provides the first demonstration that the host CypA interacts with IBDV VP4. The overexpression of CypA suppresses IBDV replication, whereas the knock-down of CypA promotes the replication of IBDV. Thus, our findings indicate that the host cell protein CypA interacts with viral VP4 and inhibits the replication of IBDV. These findings contribute to a further understanding of the molecular mechanism of IBDV infection.

## Figures and Tables

**Table 1:** Primers and siRNAs used in this study.

Primers/siRNAs	Sequences (5′-3′)	Restriction enzyme	Usage
VP4-U	AGAGAATTCGCCGACAAGGGGTACGAG	*Eco*R I	pGB-VP4
VP4-L	AAACTGCAGAGCCATGGCAAGGTGGTA	*Pst* I	
HA-VP4-U	CTGAATTCGCCGACAAGGGGTACGAG	*Eco*R I	pCAH-VP4
HA-VP4-L	GCCTCGAGAGCCATGGCAAGGTGGTA	*Xho* I	
CypA-U	AAAGAATTCATGGCCAACCCCGTCGTG	*Eco*R I	pCAF-CypA
CypA-L	ATACTCGAGCCGAGAGCTGCCCGCAGTT	*Xho* I	
pGEX-CYPA-U	GGCGAATTCGCCAACCCCGTCGTGTTC	*Eco*R I	pGEX-CypA
pGEX-CYPA-L	GCTGAGCTCCGAGAGCTGCCCGCAGTT	*Xho* I	
T7SPU	TAATACGACTCACTATAGGGC		pGADT7
3ASPL	ACACGTAGCACGTGGTAGA		
FVP5	CCTTCTGATGCCAACAAC		Real-time RT-PCR
RVP5	ACAATTAGCCCTGACCCT		
PVP5	FAM-CGGACGACACCCTGGAGAAGCA-BHQ1		
RNA#1	Sense, 5′-CCGAGUGGUUGGACGGCAATT-3′		siRNAs
	Antisense, 5′-UUGCCGUCCAACCACUCGGTT-3′		
RNA#2	Sense, 5′-ACGGCAAGACGAGCAAGCATT-3′		
	Antisense, 5′-UGCUUGCUCGUCUUGCCGUTT-3′		
RNA#3	Sense, 5′-GACGAGAACUUCAUCCUGATT-3′		
	Antisense, 5′-UCAGGAUGAAGUUCUCGUCTT-3′		
Negative control (NC)	Sense, 5′-UUCUCCGAACGUGUCACGUTT-3′		
	Antisense, 5′-ACGUGACACGUUCGGAGAATT-3′		
